# Signals of interstitial lung disease with novel antineoplastic agents in ovarian cancer: a three-database disproportionality study

**DOI:** 10.3389/fphar.2025.1682276

**Published:** 2026-01-08

**Authors:** Chenguang Yang, Xuan Song, Hongmei Sun, Min Chen, Xi Chen, Chengjiang Liu, Zhongjian Wang, Xialing Zhu

**Affiliations:** 1 Department of Gynaecology and Obstetrics, Affiliated Xuancheng Hospital of Wannan Medical College, Xuancheng, Anhui, China; 2 Department of General Medicine, Affiliated Xuancheng Hospital of Wannan Medical College, Xuancheng, Anhui, China; 3 Department of Epidemiology and Statistics, School of Public Health, Medical College, Zhejiang University, Hangzhou, China; 4 Department of General Medicine, Affiliated Anqing First People’s Hospital of Anhui Medical University, Hefei, China; 5 BCPMdata Pharma Technology (Chengdu) Co., Ltd., Chengdu, Sichuan, China

**Keywords:** adverse events, ovarian cancer, interstitial lung disease, novel antineoplastic agents, Bayesian disproportional analysis

## Abstract

**Objective:**

Emerging evidence suggests that certain antitumor drugs may be associated with interstitial lung disease (ILD); however, large-scale real-world data remain limited. This study aimed to identify disproportionate reporting signals of ILD associated with novel antineoplastic agents used in ovarian cancer.

**Methods:**

Data from the US Food and Drug Administration Adverse Event Reporting System (FAERS; 2015 - Q1 2025), the Canada Vigilance Adverse Reaction Database (CVAR; 1965 - November 2024), and the Japanese Adverse Drug Event Report Database (JADER; 2004 - Q3 2024) were analyzed. Reports involving FDA-approved novel antineoplastic agents for ovarian cancer were included. Bayesian disproportional analysis was conducted to generate the proportional reporting ratio (PRR), reporting odds ratio (ROR), and information component (IC) to detect signals.

**Results:**

Eventually, 4,089 eligible records were retrieved, in which 2,783 ILD reports were from FAERS, 334 from CVAR, and 972 from JADER. Three drugs exhibited significant ILD reporting signals in FAERS: olaparib (ROR = 3.43, IC_025_ = 1.41), bevacizumab (ROR = 1.51, IC_025_ = 0.34), and mirvetuximab soravtansine-gynx (ROR = 4.27, IC_025_ = 1.40). Olaparib also showed ILD signals in CVAR (ROR = 3.43, IC_025_ = 0.73) and JADER (ROR = 1.55, IC_025_ = 0.26). Notably, mirvetuximab soravtansine-gynx associated ILD had the highest death constituent ratio (29.41%, 5/17). Compared to chemotherapy drugs, mirvetuximab soravtansine-gynx and olaparib demonstrated stronger associations with ILD.

**Conclusion:**

Patients with ovarian cancer administrating mirvetuximab soravtansine-gynx or olaparib merit close monitor for ILD. Early detection and immediate intervention are critical.

## Introduction

From 1990 to 2021, the global incidence of ovarian cancer, fallopian tube cancer, and primary peritoneal cancer (collectively referred to as ovarian cancer) increased from 159,096 to 298,876 cases, with related deaths rising from 100,584 to 185,609 (Tianye et al., 2025). Ovarian cancer remains one of the most lethal female-specific malignancies. Although early-stage ovarian cancer usually results in moderately satisfying outcome, most patients are diagnosed at end-stage, namely III/IV, resulting in a mortality rate exceeding 75% due to the lack of early detection. For decades, cytoreductive surgery and platinum-taxane chemotherapy have been the cornerstone of ovarian cancer treatment. However, the benefit of surgery in advanced epithelial ovarian cancer remains uncertain (Harter et al., 2017). While 80% of high-grade serous carcinoma patients show modest respond to initial chemotherapy, relapse is often inevitable, necessitating the development of novel targeted therapies. Currently, FDA-approved drugs for ovarian cancer include: poly ADP - ribose polymerase (PARP) inhibitors (Olaparib, Niraparib, Rucaparib), Monoclonal antibodies (mAb) (Bevacizumab), and Antibody-drug conjugate (ADC) (Mirvetuximab soravtansine-gynx).

However, adverse drug reactions associated with these therapies have raised certain concerns. Unlike drug-induced liver or kidney injury, drug-induced lung injury has been overlooked. Drug-induced interstitial lung disease (DILD), the most severe phenotype of drug-induced lung injury, involves inflammatory and fibrotic lung damage, manifesting as acute dyspnea, fever, or progressive respiratory function decline. Chest CT findings are perplexed, including ground-glass opacities, organizing pneumonia, or diffuse alveolar damage. Due to its non-specific presentation in terms of radiological findings and symptoms, DILD is often misdiagnosed or neglected, leading to treatment delays, tumor-related therapy discontinuation, or even fatal outcomes (Skeoch et al., 2018; Zhang et al., 2021). The European Medicines Agency classifies DILD as an Important Medical Event, accentuating the need for enhanced monitoring and research (European Medicines Agency, 2025).

Mirvetuximab soravtansine-gynx, a novel folate receptor α-targeting ADC, combines a chimeric IgG1 antibody with the microtubule inhibitor DM4. In November 2022, the FDA approved it for platinum-resistant EOC based on the SORAYA trial (NCT04296890) (Dilawari et al., 2023), which excluded patients with Grade >1 non-infectious ILD. However, the MIRASOL trial (NCT04209855) reported ≥10% pneumonia incidence, with 3% of patients discontinuing treatment due to pneumonitis (Kathleen et al., 2023).

These findings suggested that randomized controlled trials (RCTs) with over-rigorous inclusive and exclusive criteria might underestimate drug-related ILD risk, on the contrary, real-world studies, with diverse clinical settings and extended follow-up, would provide more accurate assessment of adverse drug reaction incidence.

PARP inhibitors exploit the principle of synthetic lethality to selectively target cells with homologous recombination deficiencies, significantly improving progression-free survival and overall survival in ovarian cancer patients. This advancement has reshaped the landscape of oncology (Christopher and Lord, 2017). Since the FDA approved olaparib in December 2014 for ovarian cancer patients with BRCA mutations, rare adverse events—including respiratory complications—have been increasingly reported (Christopher et al., 2019). However, existing evidence primarily relies on Japanese case reports or small sample RCTs (Hiroshi et al., 2023). Although the FDA has issued warnings regarding PARP inhibitor-associated pneumonitis, large-scale, cross-regional real-world data remain scarce.

Individual case safety report databases offer valuable real-world evidence by aggregating adverse event reports, facilitating the detection of signals that RCTs may overlook, particularly those linked to ILD. Bayesian disproportionality analysis is particularly advantageous for rare events, heterogeneous populations, or large-scale datasets. When adverse event reports are sparse, this method minimizes signal distortion caused by minor numerical fluctuations, enabling a more robust assessment of drug safety in real-world settings.

Previous research on ovarian cancer treatments has largely focused on single agents or drug classes, with limited cross-category comparisons (Hiroshi et al., 2023; Masato et al., 2023; Zhichao et al., 2023). In contrast, our study is the first large-scale real-world analysis evaluating ILD signals associated with FDA-approved novel antineoplastic agents for ovarian cancer, including ADC, PARP inhibitors, and mAb. Leveraging three major pharmacovigilance databases, we obtained a comprehensive dataset to enhance result reliability. We comprehensively analyzed ILD cases related to the treatment of novel antineoplastic agents and traditional chemotherapy drugs in ovarian cancer patients, and summarized the diagnosis and treatment of drug-related ILD.

## Methods

### Data source

This disproportional analysis utilizes data from three pharmacovigilance databases: the U.S. Food and Drug Administration Adverse Event Reporting System (FAERS), the Canada Vigilance Adverse Reaction Database (CVAR), and the Japanese Adverse Drug Event Report Database (JADER). FAERS, managed by the U.S. Food and Drug Administration, is a comprehensive repository of spontaneous adverse event and medication error reports submitted by healthcare professionals, consumers, and manufacturers. The database comprises seven distinct datasets: patient demographic and administrative information, drug information, adverse events, patient outcomes, report sources, therapy start and end dates, and indications for drug use. Similarly, CVAR and JADER are maintained by Health Canada and Japan’s Pharmaceuticals and Medical Devices Agency, respectively. Both databases compile adverse drug reaction reports from healthcare providers, pharmaceutical companies, and consumers. In all three databases, adverse events are coded using the Preferred Terms of the Medical Dictionary for Regulatory Activities (MedDRA, version 27.1). Since FAERS, CVAR, and JADER consist of fully anonymized and de-identified records, this study did not require ethical approval or informed consent.

### Procedures

We collected data on novel antineoplastic agents and traditional chemotherapy drugs approved by the FDA for ovarian cancer treatment from three pharmacovigilance databases: FAERS (2015–Q1 2025), CVAR (1965–31 December 2024), JADER (2004–Q3 2024). Novel antineoplastic agents included: PARP inhibitors: olaparib, niraparib, rucaparib; Monoclonal antibodies (mAb): bevacizumab; Antibody-drug conjugates (ADC): mirvetuximab soravtansine-gynx. Traditional chemotherapy drugs included: carboplatin, cisplatin, cyclophosphamide, gemcitabine hydrochloride, doxorubicin hydrochloride, liposomal doxorubicin hydrochloride, topotecan hydrochloride, and paclitaxel. Trade names were also considered. Given the high heterogeneity of ovarian cancer pharmacotherapeutic regimens—i.e., multi-line, multi-drug combinations—we selected adverse drug event reporting patterns for drugs designated as primary suspect. To minimize duplicate reports, we followed FDA recommendations for data deduplication. Cases sharing the same “primary ID” were considered duplicates, and only the most complete report was retained. If multiple reports contained identical demographic or clinical information (e.g., age, reporting country, adverse event type, event date), the latest or most comprehensive record was included. To identify ILD-related events, we applied a narrow-definition Standardized MedDRA Query (SMQ) (code: 20000042) (Zijun et al., 2025). Data collection was based on all Preferred Terms within this SMQ (Supplementary Table S1). Reports were filtered to include only those where the “indication” field specified ovarian, fallopian tube, or primary peritoneal cancer. Cases with unclear or irrelevant indications were excluded. Using patient age, suspect drug, event date, and reporting country from FAERS, we removed duplicate cases across all three databases and performed disproportionality analysis on CVAR and JADER. To ensure methodological transparency and reproducibility, this study adhered to the READUS-PV guidelines (Fusaroli et al., 2024a; Fusaroli et al., 2024b).

### Bayesian disproportional analysis

We employed Bayesian disproportionality analysis to assess the potential association between novel antineoplastic agents and ILD in patients with ovarian cancer. Disproportionality analysis, a statistical method originally proposed by Bayes, is widely used to detect potential reporting imbalances in spontaneous reporting systems. Key statistical metrics for disproportionality analysis include the proportional reporting ratio (PRR), reporting odds ratio (ROR), and information component (IC) (Eugène et al., 2002). Higher values of these metrics indicate a stronger association between adverse events and specific drugs. A disproportional reporting signal was inferred if any of the following criteria were met: (1) the number of cases ≥3, PRR ≥2, and the chi - square value (χ^2^) ≥ 4; (2) the lower limit of the 95% confidence interval (CI) of ROR >1; (3) IC_025_ > 0. The formulas for these metrics, including χ^2^ and 95% CI, are provided below (Eugène et al., 2002).

$$\text{PRR}=\frac{a/\left(a+c\right)}{b/\left(b+d\right)}$$

$$\text{PRR }95\%\text{ CI}=e^{\ln\left(\text{PRR}\right)\pm 1.96\sqrt{\frac{1}{a}-\frac{1}{a+c}+\frac{1}{b}-\frac{1}{b+d}}}$$

$$\text{ROR}=\frac{\left(a/c\right)}{\left(b/d\right)}=\frac{\text{ad}}{\text{bc}}$$

$$\text{ROR }95\%\;\text{CI}=e^{\ln\left(R0R\right)\pm 1.96\sqrt{\frac{1}{a}+\frac{1}{b}+\frac{1}{c}+\frac{1}{d}}}$$

$$x_{\text{yates}\;}^{2}=\frac{{\left(a+b+c+d)\times (\left|\left(a\times d\right)-\left(b\times c\right)\right|-\left(a+b+c+d\right)/2\right)}^{2}}{\left(a+c\right)\times \left(a+b\right)\times \left(b+d\right)\times \left(d+c\right)}$$

$$\text{In formation component }\left(\text{IC}\right)=\log_{2}\frac{a+0.5}{a_{\exp\;}+0.5}$$

$$a_{\exp}=\frac{\left(a+b\right)\;*\;\left(a+c\right)}{\left(a+b+c+d\right)}$$

$$IC_{025}=\text{IC}-3.3\times {\left(a+0.5\right)}^{-1/2}-2\times {\left(a+0.5\right)}^{-3/2}$$

Abbreviations: a, The number of reports of the drug of interest with the adverse event of interest; b, The number of reports of all other drugs with the adverse event of interest; c, The number of reports of the drug of interest with all other adverse events; d, The number of reports of all other drugs with all other adverse events; PRR, proportional reporting ratio; ROR, reporting odds ratio; CI, confidence interval; IC, information component.

### Traditional statistical analysis

We performed a descriptive analysis of clinical characteristics. Non-normally distributed data were summarized using median and interquartile range (IQR), while categorical variables were reported as frequencies and percentages. Logistic regression was applied to compare the proportions of ILD among all adverse events by novel antineoplastic agents versus traditional chemotherapy drugs in ovarian cancer treatment. Additionally, we used logistic regression to assess differences in severe ILD incidence between drug classes. For ordinal outcomes, ordinal logistic regression was employed to compare response severity. All statistical analyses were conducted using Stata 17.0 MP. To visually compare ILD signals between novel and traditional agents, we generated a volcano plot using ROR and adjusted P-values. This plot highlights differential ILD signal strengths between the two drug categories. A two-sided *p* < 0.05 was considered statistically significant.

## Results

### Descriptive analysis

In the FAERS database, a total of 314,902 eligible records were included, among which 2,783 reports of ILD were identified. The median age of patients was 66 years (IQR: 58–71), and the median weight was 60 kg (IQR: 51.3–60.92). Serious adverse events were reported in 98.46% (2,740/2,783) of ILD cases. In the CVAR database, 43,326 eligible records were analyzed, identifying 334 ILD reports. The median age was 65 years (IQR: 56–67), and the median weight was 67 kg (IQR: 56–72.15). All ILD reports were classified as serious. In the JADER database, 21,412 eligible records were included, with 972 ILD reports identified. The median age was 60 years (IQR: 50–70), and the median weight was 50 kg (IQR: 40–50) (Table 1). Data processing procedures are illustrated in Supplementary Figure S1.

**TABLE 1:** Clinical characteristics of patients with ovarian, fallopian tube, and primary peritoneal cancer-associated interstitial lung disease in the FAERS, CVAR, and JADER databases.

Characteristics	FAERS NO. (%)	CVAR NO. (%)	JADER NO. (%)
Number of patients	2,783	334	972
Age (years)			
Data available	1,807	326	870
<30	49 (2.71%)	2 (0.61%)	43 (4.94%)
30–39	50 (2.77%)	46 (14.11%)	28 (3.22%)
40–49	99 (5.48%)	28 (8.59%)	82 (9.43%)
50–59	297 (16.44%)	69 (21.17%)	196 (22.53%)
60–69	730 (40.39%)	143 (43.87%)	282 (32.41%)
70–79	455 (25.18%)	36 (11.04%)	203 (23.33%)
≥80	127 (7.03%)	2 (0.61%)	36 (4.14%)
Median (IQR)	66 (58–71)	65 (56–67)	60 (50–70)
Weight (kg)			
Data available	909	202	512
<40	21 (2.31%)	NA	58 (11.33%)
40–49	146 (16.06%)	31 (15.34%)	192 (37.50%)
50–59	277 (30.48%)	26 (12.87%)	182 (35.55%)
60–69	239 (26.29%)	92 (45.55%)	67 (13.09%)
70–79	116 (12.76%)	42 (20.79%)	10 (1.95%)
80–89	45 (4.95%)	11 (5.45%)	3 (0.58%)
≥90	65 (7.15%)	NA	NA
Median (IQR)	60 (51.3–60.92)	67 (56–72.15)	50 (40–50)
Reported countries (Top 6)			
United States	1,008 (36.22%)	NA	NA
Japan	708 (25.44%)	NA	NA
France	304 (10.92%)	NA	NA
Italy	111 (3.98%)	NA	NA
Germany	107 (3.84%)	NA	NA
Canada	85 (3.05%)	NA	NA
Seriousness			
Serious	2,740 (98.45%)	334 (100%)	NA
Not serious	43 (1.55%)	NA	NA
Seriousness criteria			
Data available	1,089	248	​
Death	192 (17.63%)	32 (12.91%)	NA
Life-threatening	110 (10.10%)	34 (13.71%)	NA
Hospitalization	763 (70.06%)	178 (71.77%)	NA
Disability	24 (2.21%)	4 (1.61%)	NA
Outcomes			
Data available	1,710	250	810
Recovered	795 (46.49%)	13 (5.2%)	301 (37.16%)
Recovering	397 (23.22%)	3 (1.2%)	298 (36.79%)
Not recovered	302 (17.66%)	187 (74.8%)	78 (9.63%)
Recovered with sequelae	15 (0.88%)	15 (6%)	8 (0.99%)
Death	201 (11.75%)	32 (12.8%)	125 (15.43%)

Certain characteristics are reported in only one database, while the results for these categories are deemed not applicable (NA) in other databases.

Abbreviations: FAERS, FDA, adverse event reporting system; CVAR, Canada vigilance adverse reaction; JADER, Japanese adverse drug event report; IQR, interquartile range; NA, not applicable.

### Bayesian disproportional analysis

In FAERS, disproportionate reporting signals associated with ILD were identified for three novel antineoplastic agents: olaparib (PRR = 3.36, 95% CI: 3.06–3.70; χ^2^ = 682.39; ROR = 3.43, 95% CI: 3.11–3.78; IC = 1.56; IC_025_ = 1.41), bevacizumab (PRR = 1.50, 95% CI: 1.34–1.68; χ^2^ = 47.32; ROR = 1.51, 95% CI: 1.34–1.69; IC = 0.53, IC_025_ = 0.34), and mirvetuximab soravtansine - gynx (PRR = 4.15, 95% CI: 2.97–5.82; χ^2^ = 78.29; ROR = 4.27, 95% CI: 3.01–6.06; IC = 1.98, IC_025_ = 1.40). In CVAR, a disproportionate ILD signal was detected for olaparib (PRR = 3.37, 95% CI: 2.02–5.62; χ^2^ = 24.08; ROR = 3.43, 95% CI: 2.03–5.80; IC = 1.61, IC_025_ = 0.73). In JADER, also indicated a disproportionate ILD signal for olaparib (PRR = 1.51, 95% CI: 1.32–1.74; χ^2^ = 34.70; ROR = 1.55, 95% CI: 1.34–1.79; IC = 0.46, IC_025_ = 0.26).

Additionally, we combined all eligible reports of ovarian cancer from the three databases into a single dataset. This analysis revealed disproportionate ILD signals for two novel antineoplastic agents: olaparib (PRR = 3.47, 95% CI: 3.21–3.74; χ^2^ = 1106.36; ROR = 3.55, 95% CI: 3.28–3.84; IC = 1.58, IC_025_ = 1.47) and mirvetuximab soravtansine-gynx (PRR = 3.50, 95% CI: 2.50–4.90; χ^2^ = 59.15; ROR = 3.60, 95% CI: 2.54–5.10; IC = 1.75, IC_025_ = 1.17). Notably, bevacizumab showed an ILD signal in FAERS but not in FAERS + CVAR + JADER (PRR = 0.96, 95% CI: 0.87–1.06; χ^2^ = 0.65; ROR = 0.96, 95% CI: 0.87–1.06; IC = −0.05, IC_025_ = −0.20). It should be noted that Mirvetuximab soravtansine-gynx is currently only marketed in the United States, Germany, and Poland, and is not available in Canada and Japan. Therefore, there are no relevant records in CVAR and JADER (Table 2).

**TABLE 2:** Raw data used in the disproportionality analysis to calculate the ROR.

Drug	a	b	c	d	PRR (95% CI)	ROR (95% CI)	χ^2^	IC	IC025
*FAERS + CVAR + JADER*									
Novel antineoplastic agent									
Niraparib Tosylate monohydrate	607	3,482	147,133	228,418	0.27 (0.25, 0.30)	0.27 (0.25, 0.30)	1000.53	−1.39	−1.52
Olaparib	768	3,321	22,977	352,574	3.47 (3.21, 3.74)	3.55 (3.28, 3.84)	1106.36	1.58	1.47
Bevacizumab	481	3,608	45,736	329,815	0.96 (0.87, 1.06)	0.96 (0.87, 1.06)	0.65	−0.05	−0.20
Rucaparib camsylate	81	4,008	31,093	344,458	0.23 (0.18, 0.28)	0.22 (0.18, 0.28)	212.89	−2.04	−2.41
Mirvetuximab soravtansine-gynx	33	4,056	847	374,704	3.50 (2.50, 4.90)	3.60 (2.54, 5.10)	59.15	1.75	1.17
Chemotherapeutic agent									
Carboplatin	341	3,748	30,507	345,044	1.03 (0.92, 1.15)	1.03 (0.92, 1.15)	0.25	0.04	−0.14
Cisplatin	90	3,999	4,400	371,151	1.88 (1.53, 2.31)	1.90 (1.54, 2.34)	36.68	0.89	0.54
Cyclophosphamide	34	4,055	1,852	373,699	1.68 (1.20, 2.35)	1.69 (1.20, 2.38)	9.37	0.73	0.16
Gemcitabine hydrochloride	151	3,938	5,692	369,859	2.45 (2.09, 2.88)	2.49 (2.11, 2.94)	126.53	1.26	0.99
Doxorubicin hydrochloride	416	3,673	11,387	364,164	3.53 (3.19, 3.90)	3.62 (3.27, 4.02)	684.85	1.71	1.54
Doxorubicin hydrochloride liposome	16	4,073	897	374,654	1.63 (1.00, 2.65)	1.64 (1.00, 2.70)	3.92	0.68	−0.17
Topotecan hydrochloride	6	4,083	1,080	374,471	0.51 (0.23, 1.14)	0.51 (0.23, 1.14)	2.81	−0.91	−2.32
Paclitaxel	404	3,685	33,670	341,881	1.11 (1.00, 1.23)	1.11 (1.00, 1.23)	4.14	0.14	−0.03
*FAERS*									
Novel antineoplastic agent									
Niraparib tosylate monohydrate	513	2,270	138,129	173,990	0.29 (0.26, 0.32)	0.28 (0.26, 0.31)	746.34	−1.26	−1.40
Olaparib	495	2,288	18,529	293,590	3.36 (3.06, 3.70)	3.43 (3.11, 3.78)	682.39	1.56	1.41
Bevacizumab	318	2,465	24,624	287,495	1.50 (1.34, 1.68)	1.51 (1.34, 1.69)	47.32	0.53	0.34
Rucaparib camsylate	81	2,702	31,093	281,026	0.27 (0.22, 0.34)	0.27 (0.22, 0.34)	153.77	−1.76	−2.13
Mirvetuximab soravtansine-gynx	33	2,750	874	311,245	4.15 (2.97, 5.82)	4.27 (3.01, 6.06)	78.79	1.98	1.40
Chemotherapeutic agent									
Carboplatin	299	2,484	28,214	283,905	1.21 (1.07, 1.36)	1.21 (1.07, 1.37)	9.73	0.25	0.06
Cisplatin	52	2,731	3,983	308,136	1.47 (1.12, 1.93)	1.47 (1.12, 1.94)	7.65	0.54	0.08
Cyclophosphamide	27	2,756	1,789	310,330	1.70 (1.16, 2.46)	1.70 (1.16, 2.50)	7.58	0.73	0.09
Gemcitabine hydrochloride	89	2,694	5,267	306,852	1.91 (1.55, 2.35)	1.92 (1.56, 2.38)	37.64	0.90	0.55
Doxorubicin hydrochloride	177	2,606	7,451	304,668	2.74 (2.35, 3.18)	2.78 (2.38, 3.24)	184.19	1.39	1.14
Topotecan hydrochloride	6	2,777	1,080	311,039	0.62 (0.28, 1.39)	0.62 (0.28, 1.39)	1.37	−0.64	−2.05
Paclitaxel	265	2,518	23,640	288,479	1.28 (1.13, 1.45)	1.28 (1.13, 1.46)	14.92	0.33	0.12
*CVAR*									
Novel antineoplastic agent									
Niraparib tosylate monohydrate	21	313	5,684	37,308	0.44 (0.28, 0.69)	0.44 (0.28, 0.69)	13.94	−1.05	−1.78
Olaparib	15	319	581	42,411	3.37 (2.02, 5.62)	3.43 (2.03, 5.80)	24.08	1.61	0.73
Bevacizumab	108	226	19,086	23,906	0.60 (0.48, 0.76)	0.60 (0.48, 0.75)	19.53	−0.45	−0.77
Chemotherapeutic agent									
Cyclophosphamide	7	327	63	42,929	13.23 (6.50, 26.93)	14.59 (6.63, 32.09)	78.07	2.85	1.55
Doxorubicin hydrochloride	20	314	895	42,097	2.95 (1.89, 4.62)	3.00 (1.90, 4.73)	24.46	1.44	0.69
Paclitaxel	100	234	7,976	35,016	1.87 (1.48, 2.35)	1.88 (1.48, 2.37)	28.34	0.70	0.35
Doxorubicin hydrochloride liposome	16	318	897	42,095	2.34 (1.42, 3.85)	2.36 (1.42, 3.92)	11.75	1.13	0.29
*JADER*									
Novel antineoplastic agent									
Niraparib tosylate monohydrate	73	899	3,320	17,120	0.43 (0.34, 0.55)	0.42 (0.33, 0.53)	53.06	−1.07	−1.46
Olaparib	258	714	3,867	16,573	1.51 (1.32, 1.74)	1.55 (1.34, 1.79)	34.70	0.46	0.26
Bevacizumab	55	917	2,026	18,414	0.56 (0.43, 0.73)	0.55 (0.41, 0.72)	19.13	−0.77	−1.22
Chemotherapeutic agent									
Carboplatin	40	932	2,295	18,145	0.35 (0.26, 0.48)	0.34 (0.25, 0.47)	48.32	−1.39	−1.92
Cisplatin	38	934	417	20,023	1.87 (1.37, 2.56)	1.95 (1.39, 2.74)	15.59	0.86	0.32
Gemcitabine hydrochloride	62	910	425	20,015	2.92 (2.30, 3.73)	3.21 (2.44, 4.22)	77.16	1.47	1.05
Doxorubicin hydrochloride	219	753	3,041	17,399	1.62 (1.40, 1.87)	1.66 (1.42, 1.94)	42.11	0.56	0.34
Paclitaxel	39	933	2,054	18,386	0.39 (0.28, 0.53)	0.37 (0.27, 0.52)	38.34	−1.27	−1.81

Abbreviations: A, the number of reports of the drug of interest with the adverse event of interest; B, the number of reports of all other drugs with the adverse event of interest; C, the number of reports of the drug of interest with all other adverse events; D, the number of reports of all other drugs with all other adverse events; PRR, proportional reporting ratio; ROR, reporting odds ratio; CI, confidence interval; IC, information component; FAERS, FDA, adverse event reporting system; CVAR, Canada vigilance adverse reaction; JADER, Japanese adverse drug event report.

To evaluate the potential confounding effect of age on the association between novel antineoplastic agents and ILD, we stratified all reports with available age information into two age groups: patients aged ≥65 years and those <65 years. While ILD signals were consistent across age groups in both the FAERS and JADER databases, Olaparib-associated ILD signals in the CVAR database were observed exclusively in patients younger than 65 years. We hypothesize that this discrepancy may be attributable to missing age information and the resulting limited number of reports for Olaparib in patients aged 65 years and older, leading to findings divergent from the other two databases (Supplementary Table S2).

### Analysis of fatal cases

**FIGURE 1:**
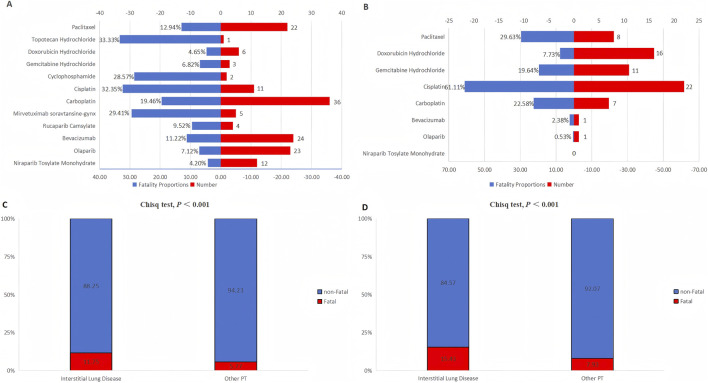
Analysis of ILD-associated fatal cases with novel antineoplastic agents. **(A,B)** Showing the fatality proportions and number for different novel antineoplastic agents in the FAERS and JADER databases, respectively. **(C,D)** Showing the comparison of fatality risk between ILD and non-ILD (other PT) patients in the FAERS and JADER databases, respectively. Statistical significance was assessed using the chi-square test. Abbreviations: Chisq, chi-square; ILD, interstitial lung disease; PT, preferred term; FAERS, FDA Adverse Event Reporting System; JADER, Japanese Adverse Drug Event Report.

In FAERS, over 10% (201/1,710) of ILD cases resulted in fatal outcomes. Among novel antineoplastic agents, mirvetuximab soravtansine-gynx exhibited the highest mortality rate (29.41%; 5/17), whereas niraparib had the lowest (4.20%; 12/286) (Figure 1A). In JADER, the proportion of fatal ILD cases was even higher (15.43%; 125/810), primarily attributed to chemotherapy drugs. Notably, cisplatin was associated with the highest mortality rate (61.11%; 22/36) (Figure 1B). FAERS and JADER demonstrated concordant trends. The risk of death was significantly elevated in the ILD group compared to the non-ILD group (FAERS: 11.75% vs. 5.77%, *p* < 0.001; JADER: 15.43% vs. 7.93%, *p* < 0.001) (Figures 1C,D).

### Comparison with chemotherapy

**FIGURE 2:**
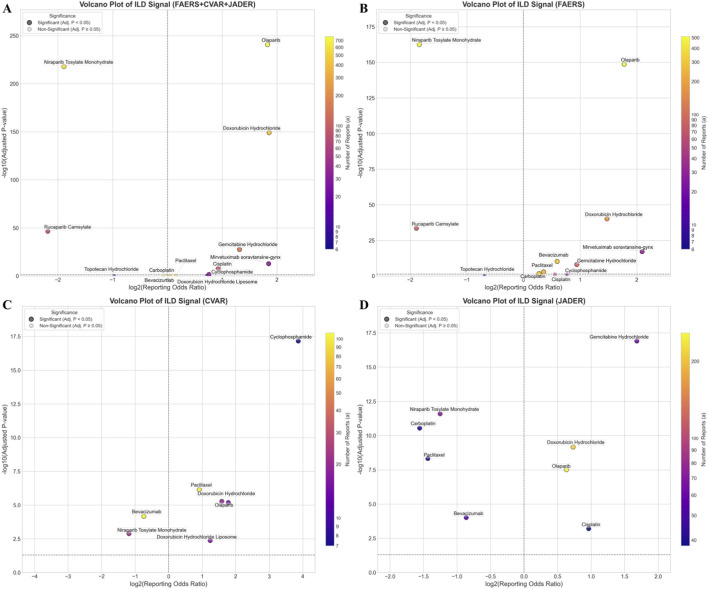
Volcano plot comparing ILD signal between novel antineoplastic agents and chemotherapeutic drugs. **(A–D)** Respectively show the comparison of novel antineoplastic agents and chemotherapy drugs in the FAERS + CVAR + JADER, FAERS, CVAR, and JADER database. The x-axis represents the logarithm of the ROR, and the y-axis represents the negative base-10 logarithm of the adjusted P value, obtained from Fisher’s exact test followed by Bonferroni correction. The colour intensity of each point indicates the number of reports, with warmer (redder) colours representing higher numbers of reports. Drugs in the upper right quadrant exhibit both higher ILD signals and significant statistical differences. Abbreviations: ROR, reporting odds ratio; ILD, interstitial lung disease; FAERS, FDA Adverse Event Reporting System; CVAR, Canada Vigilance Adverse Reaction; JADER, Japanese Adverse Drug Event Report.

In FAERS, both olaparib and mirvetuximab soravtansine-gynx showed a stronger reporting association with ILD compared to traditional chemotherapy drugs. Similar results were observed in the combined dataset composed of FAERS, CVAR, and JADER (Figure 2).

### Constituent ratio of interstitial lung disease in all adverse events caused by drug

In FAERS, a significant increase in the constituent ratio of ILD associated with mirvetuximab soravtansine-gynx and olaparib was observed compared to traditional chemotherapy drugs. In contrast, a significant decrease in the constituent ratio of ILD associated with niraparib and rucaparib was noted. Compared to other novel antineoplastic agents, a significant increase in the constituent ratio of ILD associated with mirvetuximab soravtansine-gynx and olaparib was observed, but there was no significant difference between the two. Similar results were found in the combined dataset formed by the three databases of FAERS, CVAR, and JADER. In CVAR and JADER, the constituent ratio of ILD associated with olaparib significantly increased compared to other novel antineoplastic agents (Table 3).

**TABLE 3:** Matrix of pairwise comparisons of regimens on proportion of interstitial lung disease (shown as odds ratios and 95% confidence intervals).

​	Niraparib tosylate monohydrate	Olaparib	Bevacizumab	Rucaparib camsylate	Mirvetuximab soravtansine-gynx	Carboplatin	Cisplatin	Cyclophosphamide	Gemcitabine Hydrochloride	Doxorubicin Hydrochloride	Doxorubicin Hydrochloride Liposome	Topotecan Hydrochloride	Paclitaxel
*FAERS + CVAR + JADER*													
Niraparib tosylate monohydrate	1	​	​	​	​	​	​	​	​	​	​	​	​
Olaparib	**0.12 (0.11, 0.14)**	1	​	​	​	​	​	​	​	​	​	​	​
Bevacizumab	**0.39 (0.35, 0.44)**	**3.18 (2.83, 3.57)**	1	​	​	​	​	​	​	​	​	​	​
Rucaparib camsylate	**1.58 (1.26, 2.00)**	**12.83 (10.20, 16.14)**	**4.04 (3.19, 5.11)**	1	​	​	​	​	​	​	​	​	​
Mirvetuximab soravtansine-gynx	**0.11 (0.07, 0.15)**	0.86 (0.60, 1.22)	**0.27 (0.19, 0.39)**	**0.07 (0.04, 0.10)**	1	​	​	​	​	​	​	​	​
Carboplatin	**0.37 (0.32, 0.42)**	**2.99 (2.63, 3.40)**	0.94 (0.82, 1.08)	**0.23 (0.18, 0.30)**	**3.49 (2.42, 5.01)**	1	​	​	​	​	​	​	​
Cisplatin	**0.20 (0.16, 0.25)**	**1.63 (1.31, 2.04)**	**0.51 (0.41, 0.65)**	**0.13 (0.09, 0.17)**	**1.90 (1.27, 2.86)**	**0.55 (0.43, 0.69)**	1	​	​	​	​	​	​
Cyclophosphamide	**0.22 (0.16, 0.32)**	**1.82 (1.29, 2.58)**	**0.57 (0.40, 0.81)**	**0.14 (0.09, 0.21)**	**2.12 (1.31, 3.45)**	**0.61 (0.43, 0.87)**	1.11 (0.75, 1.66)	1	​	​	​	​	​
Gemcitabine hydrochloride	**0.16 (0.13, 0.19)**	**1.26 (1.06, 1.50)**	**0.40 (0.33, 0.48)**	**0.10 (0.07, 0.13)**	**1.47 (1.00, 2.16)**	**0.42 (0.35, 0.51)**	0.77 (0.59, 1.00)	0.69 (0.48, 1.01)	1	​	​	​	​
Doxorubicin hydrochloride	**0.11 (0.10, 0.13)**	0.91 (0.81, 1.03)	**0.29 (0.25, 0.33)**	**0.07 (0.06, 0.09)**	1.07 (0.74, 1.53)	**0.31 (0.26, 0.35)**	**0.56 (0.44, 0.71)**	**0.50 (0.35, 0.72)**	**0.73 (0.60, 0.88)**	1	​	​	​
Doxorubicin hydrochloride liposome	**0.23 (0.14, 0.38)**	**1.87 (1.14, 3.09)**	**0.59 (0.36, 0.97)**	**0.15 (0.09, 0.25)**	**2.18 (1.19, 4.00)**	0.63 (0.38, 1.04)	1.15 (0.67, 1.96)	1.03 (0.57, 1.87)	1.49 (0.88, 2.50)	**2.05 (1.24, 3.39)**	1	​	​
Topotecan hydrochloride	0.74 (0.33, 1.66)	**6.02 (2.69, 13.46)**	1.89 (0.84, 4.24)	0.47 (0.20, 1.08)	**7.01 (2.92, 16.81)**	2.01 (0.90, 4.52)	**3.68 (1.61, 8.44)**	**3.30 (1.38, 7.90)**	**4.78 (2.11, 10.83)**	**6.58 (2.93, 14.76)**	**3.21 (1.25, 8.24)**	1	​
Paclitaxel	**0.34 (0.30, 0.39)**	**2.79 (2.47, 3.15)**	0.88 (0.77, 1.00)	**0.22 (0.17, 0.28)**	**3.25 (2.26, 4.66)**	0.93 (0.81, 1.08)	**1.70 (1.35, 2.15)**	**1.53 (1.07, 2.18)**	**2.21 (1.83, 2.67)**	**3.04 (2.65, 3.50)**	1.49 (0.90, 2.46)	0.46 (0.21, 1.04)	1

​	Niraparib tosylate monohydrate	Olaparib	Bevacizumab	Rucaparib camsylate	Mirvetuximab soravtansine-gynx	Carboplatin	Cisplatin	Cyclophosphamide	Gemcitabine Hydrochloride	Doxorubicin Hydrochloride	Topotecan Hydrochloride	Paclitaxel
*FAERS*												
Niraparib tosylate monohydrate	1	​	​	​	​	​	​	​	​	​	​	​
Olaparib	**0.14 (0.12, 0.16)**	1	​	​	​	​	​	​	​	​	​	​
Bevacizumab	**0.29 (0.25, 0.33)**	**2.07 (1.79, 2.38)**	1	​	​	​	​	​	​	​	​	​
Rucaparib camsylate	**1.43 (1.13, 1.80)**	**10.25 (8.10, 12.98)**	**4.96 (3.88, 6.33)**	1	​	​	​	​	​	​	​	​
Mirvetuximab soravtansine-gynx	**0.10 (0.07, 0.14)**	0.71 (0.49, 1.01)	**0.34 (0.24, 0.49)**	**0.07 (0.05, 0.10)**	1	​	​	​	​	​	​	​
Carboplatin	**0.35 (0.30, 0.40)**	**2.52 (2.18, 2.91)**	**1.22 (1.04, 1.43)**	**0.25 (0.19, 0.31)**	**3.56 (2.47, 5.14)**	1	​	​	​	​	​	​
Cisplatin	**0.28 (0.21, 0.38)**	**2.05 (1.53, 2.73)**	0.99 (0.74, 1.33)	**0.20 (0.14, 0.28)**	**2.89 (1.86, 4.50)**	0.81 (0.60, 1.09)	1	​	​	​	​	​
Cyclophosphamide	**0.25 (0.17, 0.36)**	**1.77 (1.20, 2.62)**	0.86 (0.58, 1.27)	**0.17 (0.11, 0.27)**	**2.50 (1.49, 4.19)**	0.70 (0.47, 1.04)	0.87 (0.54, 1.38)	1	​	​	​	​
Gemcitabine hydrochloride	**0.22 (0.18, 0.28)**	**1.58 (1.26, 1.99)**	**0.76 (0.60, 0.97)**	**0.15 (0.11, 0.21)**	**2.23 (1.49, 3.35)**	**0.63 (0.49, 0.80)**	0.77 (0.55, 1.09)	0.89 (0.58, 1.38)	1	​	​	​
Doxorubicin hydrochloride	**0.16 (0.13, 0.19)**	1.12 (0.95, 1.34)	**0.54 (0.45, 0.65)**	**0.11 (0.08, 0.14)**	**1.59 (1.09, 2.32)**	**0.45 (0.37, 0.54)**	**0.55 (0.40, 0.75)**	**0.64 (0.42, 0.96)**	**0.71 (0.55, 0.92)**	1	​	​
Topotecan hydrochloride	0.67 (0.30, 1.50)	**4.81 (2.14, 10.78)**	**2.32 (1.03, 5.23)**	0.47 (0.20, 1.08)	**6.80 (2.83, 16.29)**	1.91 (0.85, 4.29)	**5.67 (2.43, 13.22)**	**2.72 (1.12, 6.60)**	**3.04 (1.33, 6.97)**	**4.28 (1.89, 9.67)**	1	​
Paclitaxel	**0.33 (0.29, 0.38)**	**2.38 (2.05, 2.77)**	1.15 (0.98, 1.36)	**0.23 (0.18, 0.30)**	**3.37 (2.33, 4.87)**	0.95 (0.80, 1.12)	1.16 (0.86, 1.57)	1.35 (0.90, 2.01)	**1.51 (1.18, 1.92)**	**2.12 (1.75, 2.57)**	0.50 (0.22, 1.22)	1

​	Niraparib tosylate monohydrate	Olaparib	Bevacizumab	Cyclophosphamide	Doxorubicin Hydrochloride	Paclitaxel	Doxorubicin Hydrochloride Liposome
*CVAR*							
Niraparib tosylate monohydrate	1	​	​	​	​	​	​
Olaparib	**0.14 (0.07, 0.28)**	1	​	​	​	​	​
Bevacizumab	0.65 (0.41, 1.04)	**4.56 (2.64, 7.88)**	1	​	​	​	​
Cyclophosphamide	**0.03 (0.01, 0.08)**	**0.23 (0.09, 0.59)**	**0.05 (0.02, 0.11)**	1	​	​	​
Doxorubicin hydrochloride	**0.06 (0.03, 0.11)**	1.16 (0.59, 2.27)	**0.25 (0.16, 0.41)**	**4.97 (2.03, 12.20)**	1	​	​
Paclitaxel	**0.29 (0.18, 0.47)**	**2.06 (1.19, 3.57)**	**0.45 (0.34, 0.59)**	**8.86 (3.96, 19.83)**	**1.78 (1.10, 2.89)**	1	​
Doxorubicin hydrochloride liposome	**0.21 (0.11, 0.40)**	1.45 (0.71, 2.95)	**0.32 (0.19, 0.54)**	**6.23 (2.47, 15.70)**	1.25 (0.64, 2.43)	0.70 (0.41, 1.20)	1

​	Niraparib tosylate monohydrate	Olaparib	Bevacizumab	Carboplatin	Cisplatin	Gemcitabine Hydrochloride	Doxorubicin Hydrochloride	Paclitaxel
*JADER*								
Niraparib tosylate monohydrate	1	​	​	​	​	​	​	​
Olaparib	**0.33 (0.25, 0.44)**	1	​	​	​	​	​	​
Bevacizumab	**0.37 (0.26, 0.52)**	**2.46 (1.83, 3.30)**	1	​	​	​	​	​
Carboplatin	1.26 (0.85, 1.86)	**3.83 (2.73, 5.36)**	**1.56 (1.03, 2.35)**	1	​	​	​	​
Cisplatin	**0.24 (0.16, 0.36)**	0.73 (0.51, 1.04)	**0.30 (0.19, 0.46)**	**0.19 (0.12, 0.30)**	1	​	​	​
Gemcitabine hydrochloride	**0.15 (0.11, 0.21)**	**0.46 (0.34, 0.61)**	**0.19 (0.13, 0.27)**	**0.12 (0.08, 0.18)**	**0.62 (0.41, 0.96)**	1	​	​
Doxorubicin hydrochloride	**0.31 (0.23, 0.40)**	0.93 (0.77, 1.12)	**0.38 (0.28, 0.51)**	**0.24 (0.17, 0.34)**	1.27 (0.88, 1.81)	**2.03 (1.50, 2.73)**	1	​
Paclitaxel	1.16 (0.78, 1.71)	**3.51 (2.50, 4.94)**	1.43 (0.94, 2.16)	**0.42 (0.27, 0.65)**	**4.80 (3.03, 7.59)**	**7.68 (5.08, 11.62)**	**3.79 (2.69, 5.36)**	1

Abbreviations: FAERS, FDA, adverse event reporting system; CVAR, Canada vigilance adverse reaction; JADER, Japanese adverse drug event report.

Bolding the value indicates a statistical difference.

### Severity of reaction outcome for patients with interstitial lung disease among novel antineoplastic agents

In FAERS, compared with other novel antineoplastic agents, mirvetuximab soravtansine-gynx significantly increases the risk of more severe ILD. Compared with traditional chemotherapy drugs, other novel antineoplastic agents, except mirvetuximab soravtansine-gynx, do not show an increased risk of more severe ILD. Similar results were observed in JADER. Compared with chemotherapy drugs, novel antineoplastic agents, except doxorubicin hydrochloride, significantly reduce the risk of more severe ILD (Table 4).

**TABLE 4:** Matrix of pairwise comparisons of severity of reaction outcome for patients with interstitial lung disease (shown as odds ratios and 95% confidence intervals).

​	Niraparib tosylate monohydrate	Olaparib	Bevacizumab	Rucaparib camsylate	Mirvetuximab soravtansine-gynx	Carboplatin	Cisplatin	Cyclophosphamide	Gemcitabine hydrochloride	Doxorubicin hydrochloride	Topotecan hydrochloride	Paclitaxel
*FAERS*												
Niraparib tosylate monohydrate	1	**0.67 (0.50, 0.89)**	**0.64 (0.46, 0.89)**	0.74 (0.41, 1.35)	**3.15 (1.35, 7.36)**	0.93 (0.66, 1.31)	1.18 (0.57, 2.48)	0.38 (0.07, 2.20)	0.70 (0.40, 1.22)	**0.52 (0.35, 0.76)**	4.06 (0.57, 28.77)	0.76 (0.53, 1.07)
Olaparib	​	1	0.95 (0.68, 1.31)	1.10 (0.60, 2.00)	**4.41 (2.00, 10.12)**	1.37 (0.98, 1.92)	1.69 (0.82, 3.48)	0.56 (0.10, 3.21)	1.04 (0.60, 1.82)	0.78 (0.53, 1.14)	5.57 (0.82, 37.95)	1.12 (0.80, 1.58)
Bevacizumab	​	​	1	1.15 (0.62, 2.13)	**4.38 (1.89, 10.15)**	1.44 (0.99, 2.08)	1.75 (0.84, 3.64)	0.61 (0.11, 3.47)	1.10 (0.62, 1.96)	0.83 (0.55, 1.25)	5.49 (0.81, 37.14)	1.18 (0.81, 1.72)
Rucaparib camsylate	​	​	​	1	**3.89 (1.46, 10.33)**	1.25 (0.67, 2.32)	1.51 (0.62, 3.67)	0.52 (0.08, 3.15)	0.96 (0.45, 2.06)	0.72 (0.38, 1.37)	4.87 (0.68, 35.04)	1.02 (0.55, 1.91)
Mirvetuximab soravtansine-gynx	​	​	​	​	1	**0.32 (0.14, 0.74)**	0.39 (0.13, 1.11)	**0.13 (0.02, 0.88)**	**0.25 (0.10, 0.64)**	**0.19 (0.08, 0.44)**	1.25 (0.16, 9.71)	**0.26 (0.11, 0.62)**
Carboplatin	​	​	​	​	​	1	1.20 (0.57, 2.51)	0.41 (0.07, 2.36)	0.77 (0.43, 1.38)	**0.58 (0.38, 0.88)**	3.84 (0.57, 25.78)	0.82 (0.56, 1.21)
Cisplatin	​	​	​	​	​	​	1	0.34 (0.05, 2.15)	0.62 (0.26, 1.49)	0.47 (0.22, 1.01)	3.30 (0.43, 25.07)	0.67 (0.32, 1.42)
Cyclophosphamide	​	​	​	​	​	​	​	1	1.91 (0.31, 11.67)	1.41 (0.24, 8.24)	11.09 (0.83, 148.80)	2.06 (0.36, 11.92)
Gemcitabine hydrochloride	​	​	​	​	​	​	​	​	1	0.74 (0.40, 1.37)	5.98 (0.80, 44.50)	1.08 (0.60, 1.96)
Doxorubicin hydrochloride	​	​	​	​	​	​	​	​	​	1	**7.69 (1.08, 54.65)**	1.45 (0.94, 2.23)
Topotecan hydrochloride	​	​	​	​	​	​	​	​	​	​	1	0.21 (0.03, 1.43)
Paclitaxel	​	​	​	​	​	​	​	​	​	​	​	1

​	Niraparib tosylate monohydrate	Olaparib	Bevacizumab	Carboplatin	Cisplatin	Gemcitabine hydrochloride	Doxorubicin hydrochloride	Paclitaxel
*JADER*								
Niraparib tosylate monohydrate	1	0.81 (0.45, 1.46)	0.72 (0.32, 1.60)	**4.12 (1.78, 9.56)**	**30.18 (12.81, 71.10)**	**3.65 (1.79, 7.42)**	1.09 (0.61, 1.95)	**5.65 (2.32, 13.79)**
Olaparib	​	1	0.88 (0.45, 1.72)	**5.08 (2.48, 10.41)**	**35.98 (17.19, 75.29)**	**4.50 (2.57, 7.87)**	1.34 (0.92, 1.96)	**6.92 (3.18, 15.06)**
Bevacizumab	​	​	1	**5.25 (2.15, 12.78)**	**33.45 (13.38, 83.66)**	**4.71 (2.17, 10.20)**	1.50 (0.78, 2.90)	**6.70 (2.74, 17.84)**
Carboplatin	​	​	​	1	**6.32 (2.53, 15.79)**	0.90 (0.41, 1.98)	**0.28 (0.14, 0.57)**	1.33 (0.52, 3.44)
Cisplatin	​	​	​	​	1	**0.14 (0.06, 0.32)**	**0.04 (0.02, 0.09)**	**0.21 (0.08, 0.54)**
Gemcitabine hydrochloride	​	​	​	​	​	1	**0.32 (0.19, 0.55)**	1.46 (0.63, 3.38)
Doxorubicin hydrochloride	​	​	​	​	​	​	1	**4.26 (2.01, 9.03)**
Paclitaxel	​	​	​	​	​	​	​	1

Abbreviations: FAERS, FDA, adverse event reporting system; JADER, Japanese adverse drug event report.

Bolding the value indicates a statistical difference.

## Discussion

To our knowledge, this is the first large-sample, real-world study investigating ILD associated with novel antineoplastic agents in ovarian cancer treatment, encompassing the broadest range of drug types and the most authoritative datasets. Our findings indicated that mirvetuximab soravtansine-gynx and olaparib and bevacizumab were associated with ILD. Compared to conventional chemotherapy, both mirvetuximab soravtansine-gynx and olaparib exhibited stronger reporting associations with ILD. Notably, mirvetuximab soravtansine-gynx was linked to a significantly higher risk of severe ILD.

### Interpretation of the results by mechanisms

As the first PARP inhibitor approved by the FDA for first-line maintenance treatment of advanced ovarian cancer with BRCA gene mutations, olaparib’s common adverse drug reactions are mainly concentrated in hematological toxicity and gastrointestinal toxicity. However, with the increase in its usage, pulmonary toxicity has been successively reported in multiple studies (Yoshinobu et al., 2024; Xiaojiang et al., 2022). Zhuo et al. (2021) summarized 16 RCTs through a meta-analysis to analyze the correlation between PARP inhibitors and pneumonia. The results showed that, compared with the control treatment, PARP inhibitors significantly increased the risk of pneumonia (Peto OR 2.68 [95% CI 1.31–5.47], *p* = 0.007), and half (14/28) of the PARP inhibitor-related pneumonia events were serious adverse events. In addition, a disproportional analysis was conducted on PARP inhibitor-related pneumonia cases in the FAERS database from the first quarter of 2004 to the third quarter of 2020. The results showed that olaparib produced the most significant pneumonia signal (ROR 11.44 [8.88–14.74], IC_025_ = 2.99), which was similar to the results of our study. This consistency of results across databases further enhances the reliability of the potential association between olaparib and ILD. Olaparib may trigger ILD through cross-allergic reactions. After receiving olaparib treatment, patients presented with CT imaging findings similar to those of hypersensitivity pneumonitis (bilateral ground-glass opacities) and bronchoalveolar lavage results (alveolar lymphocytosis) (Alexandre et al., 2024; Ng et al., 2023). Of course, factors such as increasing age, the presence of underlying respiratory diseases, and the prolongation of drug excretion time due to kidney diseases may all increase the risk of drug-induced ILD. After all, with the extension of the overall survival of ovarian cancer patients, comorbidities are more common (Gemma et al., 2014; Weiner et al., 2007). It is worth noting that, by integrating three large-scale adverse reaction databases, Japan reported the most cases of drug-related ILD, which is similar to the results of previous studies (Zhichao et al., 2023). This may be related to the drug adverse event regulatory systems and racial susceptibility of different countries (Iwasa et al., 2020; Wakao et al., 2019). However, in regions other than Japan, olaparib-related ILD signals still need to be monitored.

Mirvetuximab soravtansine-gynx is a folate receptor α-directed antibody-drug conjugate with a microtubule inhibitor. In the SORAYA (NCT04296890) trial, the confirmed overall response rate was 32.4% (95% CI, 23.6–42.2), and the median duration of response was 6.9 months (95% CI, 5.6–9.7) (Ursula et al., 2023). However, mirvetuximab soravtansine-gynx related ILD cannot be ignored. The prescribing information of mirvetuximab soravtansine-gynx clearly states that patients receiving mirvetuximab soravtansine-gynx treatment may develop severe, life-threatening or fatal ILD, including pneumonia, and the incidence of drug-induced pneumonia is 10% (Elahere. Prescribing Information Package Insert, 2025). Our study, based on real-world data, fully validates the results of RCTs. It not only detects the ILD signal of mirvetuximab soravtansine-gynx but also finds that it significantly increases the risk of more severe ILD. Notably, similar results are also observed in other ADC drugs, such as trastuzumab deruxtecan and trastuzumab emtansine (Zijun et al., 2025; U.S. Food and Drug Administration, 2025). The potential mechanism by which mirvetuximab soravtansine-gynx causes pulmonary toxicity is not yet clear, but it may be related to the following points: (1) folate receptors are overexpressed in various cancer cells, including ovarian, endometrial, and breast cancer cells, but are lacking on the surface of most normal tissue cells. Although folate receptor expression can be detected in type 1 and type 2 alveolar epithelial cells and distal bronchial epithelial cells, it is mainly confined to the apical surface of polarized epithelial cells. This can effectively prevent folate receptor from being exposed to folate-drug conjugates in the bloodstream, thereby reducing the cytotoxic effect on healthy tissues (Yaxian et al., 2024). When there is damage caused by certain irritating infectious agents or environmental exposure factors, the tissue structure changes, allowing folate receptor to come into contact with mirvetuximab soravtansine-gynx in the bloodstream. This leads to apoptosis of alveolar epithelial cells, the release of inflammatory factors, and aggravation of lung damage. (2) Bystander effect: ADC drugs function by binding to targets on the tumor cell membrane to deliver cytotoxic payloads. Cells in the vicinity that do not express the target antigen can still have their cell membranes penetrated by the payload, resulting in a cytotoxic effect, which may cause damage to normal tissues. (3) Similar to other drug-induced lung injuries, exposure to any drug component or metabolite may trigger an immune-mediated hypersensitivity reaction, leading to lung damage (Osamu, 2012).

In our study, a signal of ILD associated with bevacizumab was detected in the FAERS database, but not in the CVAR and JADER databases. However, the potential risk of ILD caused by bevacizumab cannot be ignored. Currently, there is still controversy over whether bevacizumab can cause ILD. Although theoretically, the expression of vascular endothelial growth factor may increase pulmonary vascular permeability and lead to lung injury (Barratt et al., 2014). Therefore, bevacizumab, which targets vascular endothelial growth factor, may reduce the incidence of ILD. Kazuki et al. (2022) conducted a meta-analysis of 13 RCTs and revealed that compared with cancer drug treatment without bevacizumab, cancer drug treatment with bevacizumab reduced the incidence of ILD, and bevacizumab had a therapeutic effect on chemotherapy-related ILD. However, the results of a real-world study by Tianqi et al. (2022) were contrary to this. In patients with pan-cancer, the combination of bevacizumab did not reduce the risk of ILD when receiving immune checkpoint inhibitors treatment. In addition, the autopsy results of a case report clearly indicated diffuse alveolar hemorrhage after the use of bevacizumab (Satoshi et al., 2014). Of course, for the same drug, the results obtained from different adverse reaction databases may vary, which may be related to the differences in the regulatory systems of the adverse reaction databases. Yuko et al. (2022) analyzed the adverse event reports of bevacizumab submitted to the JADER database from April 2004 to March 2021. The results showed that there were 609 cases of ILD associated with bevacizumab, but the ROR = 1.06, 95% CI: 0.98–1.15.

### Analysis of fatal cases

Analysis of fatal cases shows that mirvetuximab soravtansine-gynx has a relatively high mortality rate among cases with ILD, which may be attributed to the following reasons: (1) Mirvetuximab soravtansine-gynx was approved by the FDA in November 2022 and is currently only available in the markets of the United States, Germany, and Poland. Therefore, it was not retrieved in CVAR and JADER. Similar to olaparib, after being approved by the FDA in December 2014, the number of reports on PARP inhibitor-related pneumonia in the FAERS database increased sharply from 2015 to 2019. This may be related to the “Weber effect” in the FAERS database, that is, the reporting of adverse events peaks in the initial stage after drug launch and then declines year by year (Rana et al., 2021). (2) This study adopted the SMQ for ILD, which improved the case identification rate and sensitivity by covering a wide range of clinically relevant terms. Therefore, the mortality rate observed from the database may be higher than that in previous studies. (3) Mirvetuximab soravtansine-gynx is indicated for adult patients with folate receptor α-positive, platinum-resistant epithelial ovarian cancer, fallopian tube cancer, or primary peritoneal cancer who have previously received one to three systemic treatment regimens (Dilawari et al., 2023). These patients usually have complex underlying health conditions, and DILD lacks specific clinical manifestations, and there is no consensus on the treatment approach.

In both FAERS and JADER, it was observed that the death constituent ratio of patients with ILD was significantly higher than that of patients without ILD, which is similar to the results of the study by Zijun et al. (2025). This result indicates that ILD may be a key factor leading to death and emphasizes the importance of continuous monitoring of ILD as an Important Medical Event during treatment.

### Risk factors of interstitial lung disease

Previous research results have shown that, compared with traditional chemotherapy drugs, novel antineoplastic agents are more likely to be reported to cause ILD (Michelle et al., 2024), which is also reflected in the results of our study. In addition, some non-specific risk factors need to be comprehensively evaluated at the beginning of cancer treatment, such as Japanese ethnicity, age (children and those over 60 years old), smoking history, occupational exposure history, pre-existing pulmonary lesions at baseline (especially ILD), history of pulmonary surgery, decreased respiratory function, impaired renal function, Eastern Cooperative Oncology Group performance status score ≥2, and smaller body surface area (Paolo et al., 2022).

### Identify interstitial lung disease and management

In clinical practice, it is challenging to diagnose and manage DILD caused by anticancer drugs. For patients receiving anti-cancer drugs with ILD signals but without obvious respiratory symptoms, when new lung lesions are found during routine follow-up examinations, sufficient attention should be paid. High-resolution computed tomography should be completed as early as possible, and the presence of DILD caused by anticancer drugs should always be suspected. Organizing a multidisciplinary team consultation is particularly important as it can improve the accuracy of diagnosis (Fei, 2022). Regarding whether bronchoscopy needs to be completed, according to the recommendations of the American Thoracic Society/European Respiratory Society (American Thoracic Society/European Respiratory Society. American Thoracic Society/European Respiratory Society International Multidisciplinary C onsensus Classification of the Idiopathic Interstitial PneumoniasEuropean Respiratory Society, 2002), it has no clear value in the diagnosis of ILD, but it helps to rule out other causes such as infection, alveolar hemorrhage, or tumors. The clinical management of DILD can be graded according to the extent of lung lobe involvement, clinical symptoms, or the degree of limitation in daily life (Fei, 2022). For patients with acute-onset or moderate-to-severe DILD, in addition to stopping the current anticancer drug and providing basic respiratory support (oxygen inhalation, tracheal intubation), steroids are usually used for treatment. There is currently no unified consensus or guideline on the dosage and course of treatment, and individualized treatment is required. For ADC drugs, the following treatment regimens can be referred to: For Grade 2 cases, immediately start treatment with prednisone at a dose of ≥1 mg/kg/day for ≥14 days until the clinical symptoms and chest computed tomography results are completely resolved, and then gradually reduce the dose within ≥4 weeks. For Grade 3 or higher cases, immediately start empirical high-dose intravenous injection of methylprednisolone at 500–1,000 mg/day for three consecutive days, followed by treatment with prednisone at a dose of ≥1 mg/kg/day (or an equivalent dose) for ≥14 days, or until the clinical symptoms and chest computed tomography manifestations are completely resolved, and then gradually reduce the dose within ≥4 weeks (Fei, 2022).

## Limitation

This study has several limitations. First, the data collected from the individual case safety report database are only applicable to signal detection and correlation analysis and are insufficient for causal inference. Second, the inherent selective reporting and incomplete information in the database may lead to bias. Third, the primary identification numbers used among different databases are not universal, and there are differences in reporting formats. Finally, there are cases of data omission and errors in the database, and the outcomes of some adverse event reactions are reported as unknown, which may affect the accuracy of the results.

## Conclusion

For ovarian cancer patients receiving treatment with novel antineoplastic agents, especially mirvetuximab soravtansine-gynx and olaparib, the possibility of ILD should always be suspected when there are new-onset or aggravated respiratory symptoms, or new-onset pulmonary lesions detected during routine follow-up examinations. It should be noted that Mirvetuximab soravtansine-gynx significantly increases the risk of more severe ILD. These findings can assist clinicians in timely identifying and managing ILD, thereby enhancing treatment continuity and the quality of life of patients. In fact, it is necessary to conduct larger-scale prospective studies with extended follow-up periods to confirm these research results.

## Data Availability

The original contributions presented in the study are included in the article/Supplementary Material, further inquiries can be directed to the corresponding author.
