# Supplementary material for: Signals of interstitial lung disease with novel antineoplastic agents in ovarian cancer: a three-database disproportionality study
Source: Front Pharmacol. 2026 Jan 8;16:1682276. doi: 10.3389/fphar.2025.1682276 (PMC12823856; doi:10.3389/fphar.2025.1682276)
Supplement: Supplementary file 1 [file Table1.docx]

**Table S1. The PTs included in the narrow SMQ of ILD**

| **PT** |  |  |  | **gard** | **realm** |
| --- | --- | --- | --- | --- | --- |
| Actinomycotic pulmonary infection | | | | PT | Narrow |
| Alveolitis | | | | PT | Narrow |
| Bronchiolitis | | | | PT | Narrow |
| Eosinophilic pneumonia acute | | | | PT | Narrow |
| Hypersensitivity pneumonitis | | | | PT | Narrow |
| Idiopathic pulmonary fibrosis | | | | PT | Narrow |
| Immune-mediated lung disease | | | | PT | Narrow |
| Immune-mediated pneumonitis | | | | PT | Narrow |
| Interstitial lung disease | | | | PT | Narrow |
| Leukaemic infiltration pulmonary | | | | PT | Narrow |
| Lung infection | | | | PT | Narrow |
| Lung infiltration | | | | PT | Narrow |
| Lung opacity | | | | PT | Narrow |
| Obliterative bronchiolitis | | | | PT | Narrow |
| Organising pneumonia | | | | PT | Narrow |
| Pleuroparenchymal fibroelastosis | | | | PT | Narrow |
| Pneumonitis | | | | PT | Narrow |
| Pulmonary fibrosis | | | | PT | Narrow |
| Pulmonary septal thickening | | | | PT | Narrow |
| Pulmonary toxicity | | | | PT | Narrow |
| Radiation pneumonitis | | | | PT | Narrow |
| Restrictive pulmonary disease | | | | PT | Narrow |

Abbreviations: PT, preferred term; SMQ, standardized MedDRA query; ILD, interstitial lung disease.

**Table S2. Raw data used in the disproportionality analysis to calculate the ROR (Use 65 years of age as the threshold).**

| **Drug** |  |  | **a** | **b** | **c** | **d** | **PRR (95% CI)** | | **ROR (95% CI)** | | **χ2** | **IC** | **IC025** |
| --- | --- | --- | --- | --- | --- | --- | --- | --- | --- | --- | --- | --- | --- |
| ***FAERS*** |  |  |  |  |  |  |  |  |  |  |  |  |  |
| ***Age≥65*** |  |  |  |  |  |  |  |  |  |  |  |  |  |
| Niraparib Tosylate Monohydrate |  |  | 105 | 867 | 26703 | 51964 | 0.24(0.20,0.29) |  | 0.24(0.19,0.29) |  | 230.27 | -1.64 | -1.96 |
| Olaparib |  |  | 150 | 822 | 2903 | 75764 | 4.58(3.86,5.43) |  | 4.76(3.99,5.69) |  | 359.07 | 1.99 | 1.72 |
| Bevacizumab |  |  | 138 | 834 | 8567 | 70100 | 1.35(1.13,1.61) |  | 1.35(1.13,1.62) |  | 10.79 | 0.38 | 0.09 |
| Rucaparib Camsylate |  |  | 29 | 943 | 8577 | 70090 | 0.25(0.18,0.37) |  | 0.25(0.17,0.36) |  | 62.47 | -1.84 | -2.46 |
| Mirvetuximab soravtansine-gynx |  |  | 8 | 964 | 175 | 78492 | 3.60(1.82,7.12) |  | 3.72(1.83,7.52) |  | 15.11 | 1.64 | 0.42 |
| ***Age＜65*** |  |  |  |  |  |  |  |  |  |  |  |  |  |
| Niraparib Tosylate Monohydrate |  |  | 65 | 770 | 26274 | 64841 | 0.21(0.16,0.27) |  | 0.21(0.16,0.27) |  | 179.40 | -1.87 | -2.28 |
| Olaparib |  |  | 129 | 706 | 4175 | 86940 | 3.72(3.09,4.48) |  | 3.81(3.15,4.60) |  | 218.99 | 1.71 | 1.42 |
| Bevacizumab |  |  | 112 | 723 | 9010 | 82105 | 1.41(1.15,1.71) |  | 1.41(1.16,1.72) |  | 11.50 | 0.43 | 0.12 |
| Rucaparib Camsylate |  |  | 13 | 822 | 8440 | 82675 | 0.16(0.09,0.27) |  | 0.15(0.09,0.27) |  | 58.86 | -2.52 | -3.46 |
| Mirvetuximab soravtansine-gynx |  |  | 5 | 830 | 186 | 90929 | 2.89(1.22,6.89) |  | 2.94(1.21,7.18) |  | 6.22 | 1.30 | -0.26 |
| ***CVAR*** |  |  |  |  |  |  |  |  |  |  |  |  |  |
| ***Age≥65*** |  |  |  |  |  |  |  |  |  |  |  |  |  |
| Niraparib Tosylate Monohydrate |  |  | 12 | 153 | 3171 | 12611 | 0.31(0.17,0.57) |  | 0.31(0.17,0.56) |  | 16.8 | -1.42 | -2.40 |
| Olaparib |  |  | 2 | 163 | 81 | 15701 | 2.35(0.59,9.30) |  | 2.38(0.58,9.76) |  | 0.49 | 0.88 | -1.71 |
| Bevacizumab |  |  | 52 | 113 | 7277 | 8505 | 0.54(0.39,0.75) |  | 0.54(0.39,0.75) |  | 14.00 | -0.54 | -1.00 |
| ***Age＜65*** |  |  |  |  |  |  |  |  |  |  |  |  |  |
| Niraparib Tosylate Monohydrate |  |  | 8 | 153 | 2207 | 21650 | 0.51(0.25,1.05) |  | 0.51(0.25,1.05) |  | 3.50 | -0.85 | -2.07 |
| Olaparib |  |  | 8 | 153 | 202 | 23655 | 5.93(2.95,11.91) |  | 6.12(2.97,12.63) |  | 31.35 | 2.16 | 0.94 |
| Bevacizumab |  |  | 56 | 105 | 10626 | 13231 | 0.67(0.48,0.92) |  | 0.66(0.48,0.92) |  | 6.17 | -0.35 | -0.80 |
| ***JADER*** |  |  |  |  |  |  |  |  |  |  |  |  |  |
| ***Age≥65*** |  |  |  |  |  |  |  |  |  |  |  |  |  |
| Niraparib Tosylate Monohydrate |  |  | 34 | 205 | 1043 | 3487 | 0.57(0.40,0.81) |  | 0.55(0.38,0.80) |  | 10.05 | -0.66 | -1.23 |
| Olaparib |  |  | 88 | 151 | 945 | 3585 | 2.11(1.64,2.72) |  | 2.21(1.68,2.90) |  | 34.08 | 0.76 | 0.41 |
| Bevacizumab |  |  | 12 | 227 | 305 | 4225 | 0.74(0.42,1.31) |  | 0.73(0.41,1.32) |  | 1.07 | -0.39 | -1.37 |
| ***Age＜65*** |  |  |  |  |  |  |  |  |  |  |  |  |  |
| Niraparib Tosylate Monohydrate |  |  | 26 | 605 | 1707 | 11270 | 0.29(0.20,0.43) |  | 0.28(0.19,0.42) |  | 44.19 | -1.61 | -2.27 |
| Olaparib |  |  | 120 | 511 | 1975 | 11002 | 1.29(1.06,1.57) |  | 1.31(1.07,1.61) |  | 6.66 | 0.30 | 0.01 |
| Bevacizumab |  |  | 31 | 600 | 1161 | 11816 | 0.54(0.38,0.77) |  | 0.53(0.36,0.76) |  | 12.25 | -0.82 | -1.42 |
| Abbreviations: A = The number of reports of the drug of interest with the adverse event of interest; B = The number of reports of all other drugs with the adverse event of interest; C = The number of reports of the drug of interest with all other adverse events; D = The number of reports of all other drugs with all other adverse events; PRR = proportional reporting ratio; ROR = reporting odds ratio; CI = confidence interval; IC = information component; FAERS, FDA Adverse Event Reporting System; CVAR, Canada Vigilance Adverse Reaction; JADER, Japanese Adverse Drug Event Report. | | | | | | | | | | | | | |
|  |  |  |  |  |  |  |  |  |  |  |  |  |  |
|  |  |  |  |  |  |  |  |  |  |  |  |  |  |
|  |  |  |  |  |  |  |  |  |  |  |  |  |  |
|  |  |  |  |  |  |  |  |  |  |  |  |  |  |
