# Supplementary figures and images for: Signals of interstitial lung disease with novel antineoplastic agents in ovarian cancer: a three-database disproportionality study
Source: Front Pharmacol. 2026 Jan 8;16:1682276. doi: 10.3389/fphar.2025.1682276 (PMC12823856; doi:10.3389/fphar.2025.1682276)

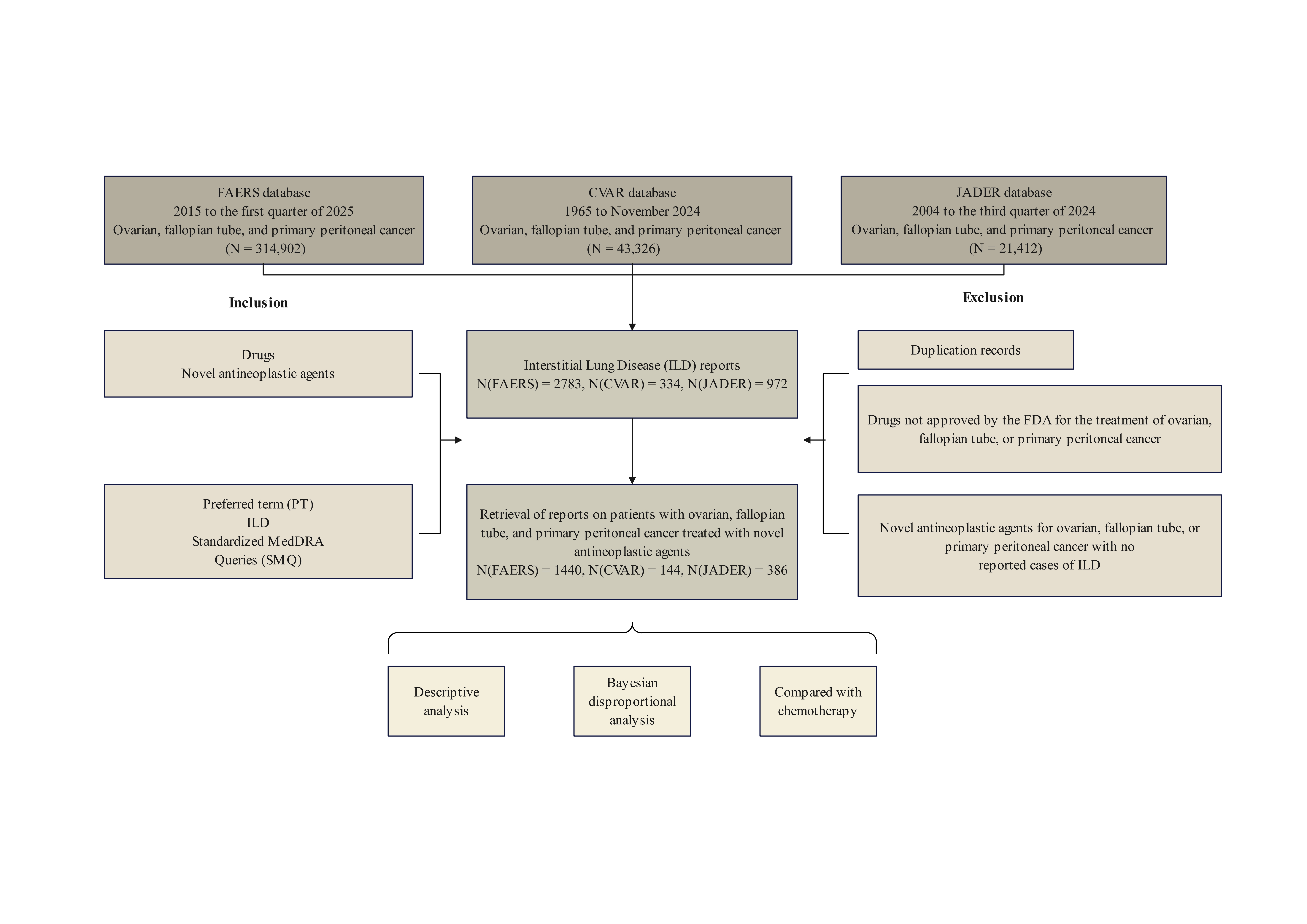

Supplement: Supplementary file 2 [file Image1.jpeg]
